# C-SH2 point mutation converts p85β regulatory subunit of phosphoinositide 3-kinase to an anti-aging gene

**DOI:** 10.1038/s41598-019-48157-6

**Published:** 2019-09-03

**Authors:** Yoshio Kano, Fukumi Hiragami, Hirotoshi Motoda, Junichi Akiyama, Yoshihisa Koike, Yutaka Gomita, Shigeki Inoue, Akihiko Kawaura, Tomohisa Furuta, Kenji Kawamura

**Affiliations:** 1 Graduate School of Health Science, Kibi International University, 8-Iga-machi, Takahashi, Okayama, 716-8508 Japan; 2 Research Institute of Health and Welfare, Kibi International University, 8-Iga-machi, Takahashi, Okayama, 716-8508 Japan; 3 Department of Occupational Therapy, Faculty of Health and Welfare, Prefectural University of Hiroshima, Mihara City, Hiroshima 723-005 Japan; 4 Department of Pharmacy, Okayama University Hospital Pharmacy, 2-5-1 Shikata-cho, Okayama, 700-8558 Japan

**Keywords:** Extracellular signalling molecules, Growth factor signalling

## Abstract

Insulin interacts with the insulin receptor, and the activated receptor promotes activity of the phosphoinositide-3 kinase (PI3K) enzyme. A decrease in insulin or insulin-like growth factor 1 (IGF-1) signaling increases the lifespan in mammalian species. We found that a point mutation in the C-SH2 domain of the p85β regulatory subunit of PI3K results in a prolonged lifespan. In p85β mutant cells, nerve growth factor (NGF) activates the longevity protein FOXO, and the mutant p85β gene produces strong resistance to oxidative stress, which contributes to aging. The p85β gene mutation causes increased serum insulin and low blood glucose in p85β mutant transgenic mice. Our results indicate that the p85β mutant allele alters the activity of downstream targets of PI3K by NGF and platelet-derived growth factor (PDGF) but not by insulin. We report that a point mutation in the C-SH2 domain of p85β transforms p85β into a novel anti-aging gene by abnormally regulating PI3K.

## Introduction

Phosphoinositide 3-kinase (PI3K) activity is induced by growth factors such as platelet- derived growth factor (PDGF), epidermal growth factor (EGF), fibroblast growth factor (FGF), nerve growth factor (NGF), and insulin. PI3K activity is required for production of the membrane lipid phosphatidylinositol-3-phosphate^1–3^. PI3K lipid products can activate various intracellular signaling pathways, including the Akt protein kinase pathway that negatively regulates Forkhead box O (FOXO), controlling lifespan^4–6^. PI3Ks are divided into classes I, II and III according to their primary structure and substrate specificity. Class II PI3K has no regulatory subunits and shows enzyme activity as a monomer, while class I PI3K is a heterodimer composed of a p110 catalytic subunit and a p85 or p55 regulatory subunit^7,8^. The catalytic subunits p110α, β, and δ are associated with the p85α, p85β, and p55γ adaptor proteins, which mediate activation through protein tyrosine kinase receptors^2^. Upon stimulation by growth factors, the p85 subunit recruits a catalytic subunit to the membrane by utilizing its SH2 domains and the phosphotyrosine motifs on activated receptor tyrosine kinases (RTKs)^9,10^.

We identified a mutation with an abnormal PI3K/Akt signaling pathway in PC12m321 cells. We found PC12m321 cells while studying neural differentiation and apoptosis using PC12m3 cells (see Cells and cell culture of Materials and Methods). Since PC12m321 cells have high resistance to stress such as H2O2 and heat shock, when we examined Akt activity, which works to suppress apoptosis, high Akt activity was observed in both PC12m3 cells and PC12m321 cells. On the other hand, PC12m321 cells showed high Akt activity in the presence of insulin but low Akt activity in the presence of NGF. High Akt activity was observed in PC12m3 cells in both the presence of insulin and the presence of NGF, and the reason for this difference was not apparent. Both insulin and NGF activate Akt via PI3K; however, the receptors that activate PI3K are different in the presence of NGF and the presence of insulin. In the case of insulin, PI3K is activated by the binding of PI3K to insulin receptor-bound IRS-1, whereas in the case of NGF, PI3K binds directly to the NGF receptor and activates PI3K. These findings suggest that PI3K may be mutated in PC12m321 cells and cannot correctly bind to the NGF receptors. The SH2 domains of the p85 regulatory subunit of PI3K attach to phosphotyrosine remnants in the context of YXXM or YMXM motifs of growth factor receptors^11^. The p85α and p85β regulatory subunits both function in several types of cells. This suggests that the mutation may be present in either p85α or p85β in PC12m321 cells, leading to the inability of PI3K to bind correctly to the NGF receptor. Firstly, the nucleotide sequence of the p85α gene was examined, but no mutation was found. However, in the p85β gene, one mutation specific to PC12m321 cells was found. Therefore, in this study, we prepared transgenic mice by incorporating the mutated p85β gene to subsequently investigate its effect on the lifespan of mice. The mutation corresponds to the binding pocket for the NGF receptor (TrkA) in the C-SH2 domain of the p85β regulatory subunit gene of class I PI3K, resulting in replacement of a lysine with an arginine. Akt, a serine/threonine protein kinase, is a key downstream mediator of PI3K signaling^12^ and negatively regulates FOXO.

## Results

**Figure 1:**
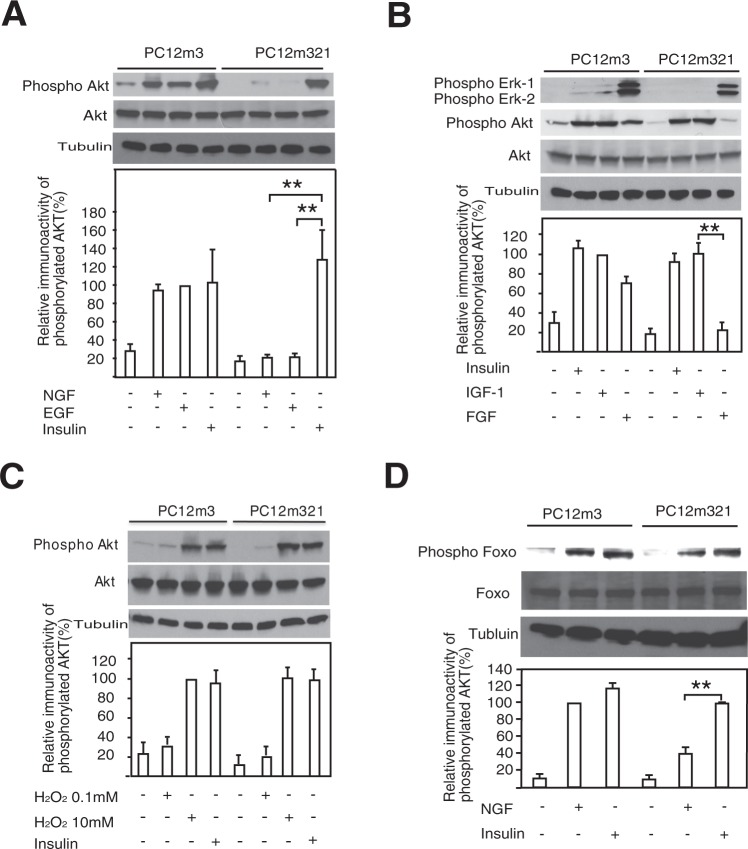
Activation of Akt and FOXO in PC12 cells by various growth factors and oxidative stress. PC12m3 and PC12m321 cells were treated with NGF (**A**), EGF (**A**), FGF (**B**), IGF-1 (**B**), insulin (**A**–**D)**, or 0.1 mM H_2_O_2_ for 120 min and with 10 mM H_2_O_2_ for 10 min to produce oxidative stress (**C**). The cell extracts were subjected to immunoblotting with anti-phospho-Akt (**A** to **C**), anti-phospho-Erk (**B**), anti-phospho-FOXO (**D**), anti-Akt, and anti-FOXO antibodies. Data are means ± s.e.m. **P < 0.01, t-test.

Serum-starved PC12m3 cells^13^ and PC12m321 cells were treated with NGF, EGF, FGF, insulin, and IGF-1. Phosphorylated Akt (pSer-473) was assayed in total cell lysates at various times after treatment (Fig. 1A,B). An increase in Akt phosphorylation was detected in PC12m3 cells and PC12m321 cells after stimulation for 30 min with NGF, EGF, FGF, insulin, and IGF-1, but the responses to NGF, EGF, and FGF were weak or absent in PC12m321 cells. An increase in Akt phosphorylation was also detected in both PC12m3 and PC12m321 cells after 10-min treatment with 10 mM H_2_O_2_ to induce oxidative stress (Fig. 1C). Thus, the mutant p85β gene in PC12m321 cells prevented PI3K/Akt signaling when NGF, EGF and FGF were used to trigger the signaling. However, the mutant p85β gene could not prevent signaling triggered by insulin, IGF-1, or oxidative stress. The growth factor-stimulated PI3K/Akt pathway negatively regulates FOXO by phosphorylation. Strong FOXO phosphorylation induced by NGF was detected in PC12m3 cells, but PC12m321 cells exhibited weak phosphorylation in response to NGF (Fig. 1D).

**Figure 2:**
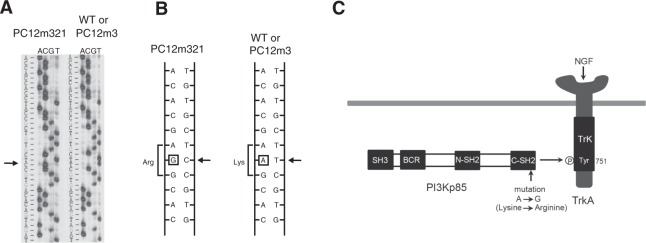
PI3K p85β gene mutation in PC12m321 cells. Sequencing gel shows a point mutation in the antisense strand (**A**). The arrowhead indicates that a thymine-to-cytosine transition occurred at nucleotide position 1979 (A → G in the sense strand, **B**). (**C**) Binding of p85 protein to the tyrosine phosphorylation site of the activated NGF receptor.

NGF-, EGF-, and FGF-induced PI3K/Akt signaling was activated weakly in PC12m321 cells. Akt is a major downstream component of signaling by PI3K, which consists of a p110 catalytic subunit and a p85 regulatory subunit. Insulin- or IGF-1-induced Akt activity was normal in PC12m321 cells, indicating that the p110 catalytic subunit is normal but that the p85 subunit is defective when NGF, EGF, or FGF are the activating ligands. Therefore, we examined the p85 gene sequence to identify the mutation in p85 protein responsible for the defective NGF response in PC12m321 cells. There was no alteration from wild-type sequences of the *pik3r1* gene (p85α) in PC12m3 or PC12m321 cells, but the *pik3r2* gene (p85β) in PC12m321 cells contained two transversion mutations: an A → G mutation (Q223R) at position 668, which was also observed in PC12m3 cells, and an A → G mutation (K660R) at position 1979, observed only in PC12m321 cells (Fig. 2A,B). Both p85α and p85β regulatory subunits of PI3K have two SH2 domains capable of binding to proteins containing phosphotyrosyl motifs (YXXM)^14^ (Fig. 2C). Lysine 660 is in the fourth β sheet (βD) of the C-terminal SH2 (c-SH2) domain of p85β and is important for binding phosphate in the phosphotyrosine pocket (Supplementary Fig. 1A,B).

**Figure 3:**
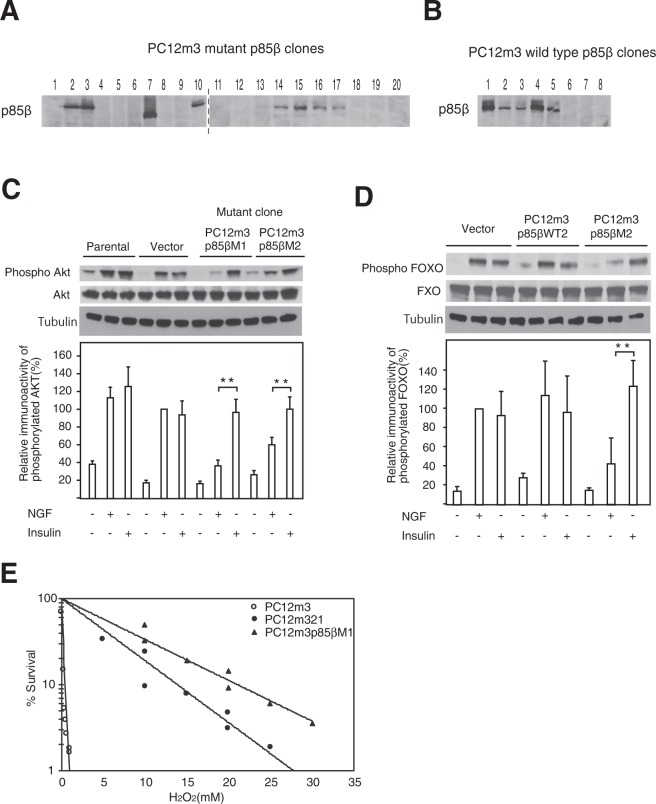
Transfection of p85β mutant gene converted PC12m3 cells to PC12m321-type cells. The protein expression levels of FLAG-epitope-tagged wild-type p85β- or mutant p85β-transfected PC12m3 cell clones including PC12m3p85βWT1 (**B**, lane 1), PC12m3p85βWT2 (**B**, lane 4), PC12m3p85βM1 (**A**, lane 2) and PC12m3p85βM2 (**A**, lane 3) were determined by immunoblotting with an anti-FLAG antibody. Clone cells in lane 7 probably have a C-terminal truncation or internal deletion of p85β protein in mutant p85β-transfected PC12m3 cells. The dividing lines of the blots indicate that they are different gels (**A**). FLAG-epitope-tagged wild-type p85β- and mutant p85β-transfected rat fibroblasts of primary cultures were selected with G418, and propagated clone cells were used for experiments on Akt and FOXO activation and survival. The levels of NGF-induced Akt phosphorylation in two mutant p85β-transfected PC12m3 cell clones were examined (**C**). The levels of NGF-induced FOXO phosphorylation in FLAG-epitope-tagged wild-type p85β- and mutant p85β-transfected PC12m3 cell clones were determined (**D**). (**E**) Effects of oxidative stress on the survival of PC12m3 and PC12m321 cells. PC12m3 (○), PC12m321 (●) and a mutant p85β-transfected PC12m3 cell clone (∆) cells were treated with H_2_O_2_ for 10 min at various concentrations ranging from 0.1 mM to 30 mM. Data are means ± s. e. m. **P < 0.01, t-test.

We next subcloned full-length wild-type p85β and mutant p85β into the EcoR1 site of the mammalian expression vector pcDNA3.1 (−) to examine the effect of p85β mutant expression on PC12m3 cells and normal human diploid fibroblasts (NHDF). A plasmid DNA with a double mutant of Q223R and K660R (p85β M) from PC12m321 cells as the p85β mutant, a single mutant of Q223R with wild-type 660 (p85β WT2) from PC12m3 cells as the p85β control, and a wild type of both 223 and 660 (p85β WT1) from PC12 cells as the p85β control were transfected into PC12m3 cells and NHDF expressing endogenous wild-type p85β, and clones were obtained by selection with the antibiotic G418 (400 μg/mL) (Fig. 3A,B). NGF-induced Akt phosphorylation levels were much lower in each of the mutant p85β-transfected PC12m3 cell lines than in PC12m3 cells and vector-transfected PC12m3 cells (Fig. 3C). PC12m3p85βM2 cells expressing mutant p85β exhibited strong FOXO phosphorylation in response to insulin but weak phosphorylation in response to NGF (Fig. 3D). On the other hand, wild-type p85β-transfected PC12m3 cells (PC12m3p85βWT2 cells) exhibited high levels of FOXO phosphorylation in response to NGF and similar levels in response to insulin. To determine the effect of altering the phosphotyrosine pocket of the C-SH2 domain of mutant p85β, we monitored the effect of oxidative stress on the survival of mutant p85β-expressing PC12m3 cells. Acute severe conditions of oxidative stress such as exposure to 1, 10, and 30 mM H_2_O_2_ for 10 min had a greater toxic effect on PC12m3 cells than on PC12m321 cells (Fig. 3E). These results indicate that PC12m321 cells had greater resistance to oxidative stress than did PC12m3 cells. When PC12m3 cells were treated with 0.3 mM H_2_O_2_ for 10 min, 85% of the cells died. In contrast, only 80% of the PC12m321 cells died even when the cells were treated with 10 mM H_2_O_2_ for 10 min. Thus, PC12m321 cells were about 30-fold more resistant than PC12m3 cells to H_2_O_2_ (Fig. 3E). Mutant p85β-expressing PC12m3 cells (PC12m3p85βM2) exhibited strong resistance to acute severe conditions of oxidative stress by treatment with H_2_O_2_ for 10 min at concentrations ranging from 0.1 to 30 mM (Fig. 3E). PC12m3p85βM2 cells exposed to a prolonged low level of oxidative stress by treatment with H_2_O_2_ for 120 min at concentrations ranging from 0.05 to 0.3 mM also showed a relatively high survival rate (Supplementary Fig. 2). Since p85β has a weak inhibitory effect on p110, it works to activate cell proliferation and carcinogenesis. We therefore examined how a lysine to arginine mutation of p85β acts on cell proliferation. We found that the proliferation of PC12m321 cells with mutant p85β was greatly suppressed compared to that of PC12 parental cells. We also found that proliferation of mutant p85β-expressing PC12m3 cells (PC12m3p85βM1 and M2) was significantly suppressed compared to that of wild-type p85β-transfected PC12m3 cells (PC12m3 p85βWT1 and WT2) (Supplementary Fig. 3).

**Figure 4:**
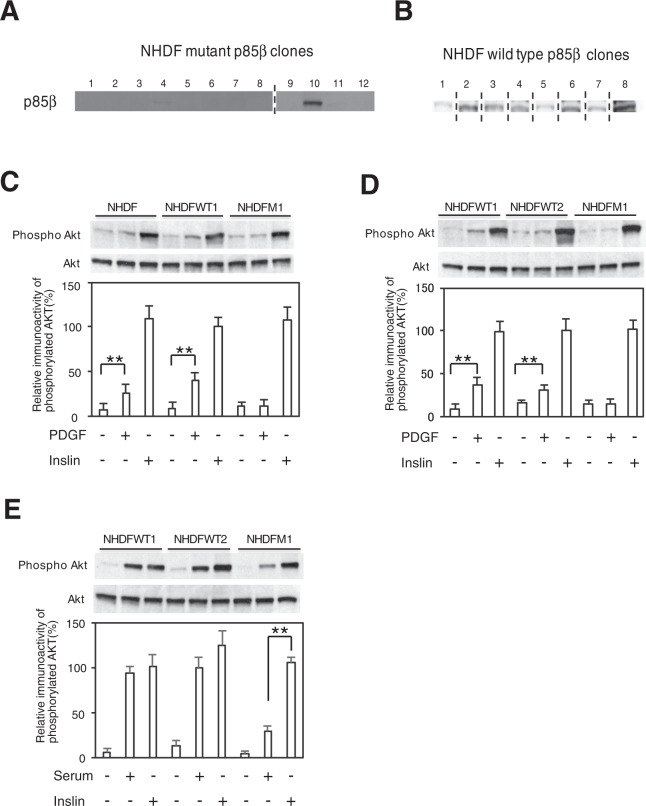
Effects of p85β mutation on normal human fibroblasts. The expression of transfected mutant p85β or wild-type p85β genes in normal human diploid fibroblasts (NHDFs) was examined. The protein expression levels of FLAG-epitope-tagged mutant (M) p85β- and wild-type (WT) p85β-transfected NHDF clones including NHDFM1, NHDFM2, NHDFWT1, and NHDFWT2 were determined by immunoblotting with an anti-FLAG antibody. The dividing lines of the blots indicate that they are different gels (**A** and **B**). Levels of PDGF (**C** and **D**)- and serum (**E**)-induced Akt phosphorylation in two wild-type p85β-transfected human fibroblast clones (NHDFWT1, NHDFWT2) and a mutant p85β-transfected human fibroblast clone (NHDFM1) were examined. Data are means ± s. e. m. **P < 0.01, t-test.

We next decided to examine the effect of p85β mutant expression on normal human diploid fibroblasts. A double mutant of Q223R and K660R was tested as the p85β mutant. Controls used were Q223R with wild-type 660 (WT2) and 223 wild type with 660 wild type (WT1). After transfection of the p85β plasmid, we obtained several clones by treatment with the antibiotic G418 (Fig. 4A,B). PDGF- or serum-induced Akt phosphorylation levels were much lower in the transfected human fibroblast clone cells with mutant p85β (Q223R and K660R) than in the transfected human fibroblast clone cells with p85β controls (WT1 and WT2) (Fig. 4C–E). The results of these human cell experiments suggest that a point mutation in the p85β gene (K660R) also converts normal human cells to the PC12m321 cell type.

**Figure 5:**
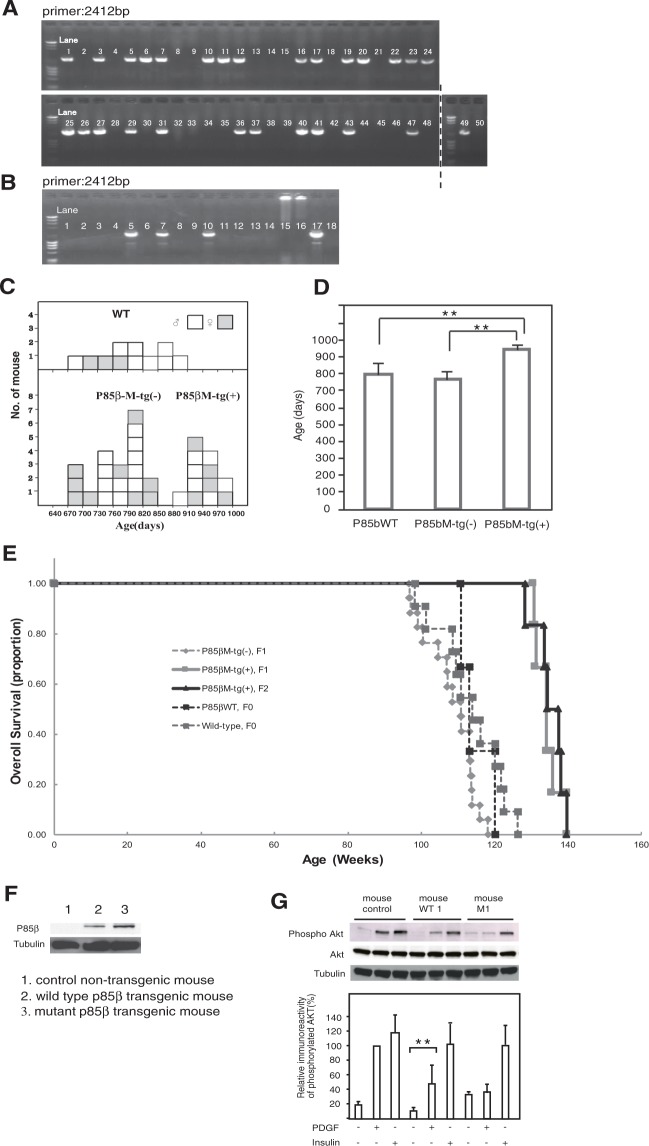
p85β mutant gene prolonged the lifespans of mice, and regulated PI3K/Akt pathways of transgenic mouse cells. The mutant and wild-type p85β transgenes were excised from their plasmids by SalI and SfoI digestion and microinjected into fertilized C57BL/6 mouse eggs. The presence of the mutant p85β transgene was examined by polymerase chain reaction of mouse genomic DNA isolated from ear punch specimens using a CMV-specific primer and rat p85β primer as described in “Materials and Methods”. Among 48 p85β mutant transgenic F1 mice, 26 were transgene-positive. Nos 49 and 50 indicate positive and negative controls of the p85β transgene. The dividing lines of the blots indicate that they are different gels (**A**). Among 16 wild-type p85β transgenic F0 mice, 3 were transgene-positive. No. 17 and 18 indicate positive and negative controls of the p85β transgene (**B**). (**C**) A total of 23 mutant p85β-transgene-positive mice were separated into a group of mice with normal lifespan (p85βM-tg(−), 17 mice) and a group of mice with a long lifespan (p85βM-tg(+), 6 mice). All of the 6 F2 mice from the p85βM-tg(+) F1 mouse showed an extended lifespan. Therefore, a total of 12 mice showed an extended lifespan. All 3 wild-type p85β-transgene-positive mice (p85βWT) showed a normal lifespan. (**D**) p85βM-tg(+) mice showed an extension of lifespan by 18.8%. *P < 0.05 (♀), **P < 0.01 (♂), t-test. (**E**) Survival curves for mutant p85β transgenic mice (total of 12 mice including 6 F1 and 6 F2 mice) and control mice (total of 37 mice including 11 WT, 23 p85βM-tg(−) and 3 p85βWT mice). (**F**) Protein expression levels of FLAG-epitope-tagged wild-type p85β- and mutant p85β transgenes of primary skin fibroblasts from transgenic mice. 1 indicates a control non-transgenic mouse, 2 indicates a wild-type p85β transgenic mouse (mouse WT1), and 3 indicates a mutant-type p85β transgenic mouse (mouse M1). (**G**) PDGF-induced-Akt phosphorylation in primary skin fibroblasts from a wild-type p85β transgenic mouse (mouse WT1) and mutant-type p85β transgenic mouse (mouse M1) was examined. Data are means ± s.e.m. **P < 0.01, t-test.

We evaluated the lifespans of transgenic mice into which the mutant p85β gene had been incorporated. The mutant and wild-type p85β transgenes were excised from their plasmids by SalI and SfoI digestion (Supplementary Fig. 4) and microinjected into fertilized C57BL/6 mouse eggs. The presence of the mutant p85β transgene was examined by polymerase chain reaction of mouse genomic DNA isolated from ear punch specimens using a CMV-specific primer and rat p85β primer as described in “Materials and Methods”. Using the minimum number of C57BL/6 mice necessary, we performed artificial insemination with mutant p85β transgene-positive F0 male mice sperm and wild-type (WT) female mice ova, obtaining 48 F1 offspring. We identified 26 (15 males and 11 females) transgene-positive individuals (Fig. 5A). Three female mice died because of an animal care accident. The other 23 mice could be separated into a group with normal lifespan (p85βM-tg(−), 17 mice) and a group with long lifespan (p85βM-tg(+), 6 mice). Although it has been reported that only male sirtuin transgenic mice have a long lifespan, mutant p85β mice demonstrated a long lifespan regardless of sex (4 males and 2 females). All of the F2 mice (4 males and 2 females) from the p85βM-tg(+) F1 mouse showed an extended lifespan. In total, 12 mice showed an extended lifespan (Fig. 5C). In the case of transgenic mice overexpressing wild-type p85β, 3 out of 16 F0 mice were transgene-positive (Fig. 5B). All 3 wild-type p85β-transgene-positive female mice showed a normal lifespan and were added to the histogram in p85βM-tg(−) (17 + 3 = 20 mice) (Fig. 5C). The mutant p85β transgenic mice (p85βM-tg(+)) showed an 18.8% lifespan extension (*P < 0.05 (♀), **P < 0.01 (♂), t-test) (Fig. 5D). Figure 5E shows survival curves for mutant p85β transgenic mice (12 mice including 6 F1 mice and 6 F2 mice) and control mice (total of 37 mice including 11 WT, 23 p85βM-tg(−) and 3 p85βWT mice). A comparison of the two resulting life table curves showed a statistically significant difference using the log-rank sum test (X^2^ = 20.1, P < 0.0001) (Fig. 5E). The p85β WT transgenic mice (p85β WT, F0) did not show a sigmoidal curve. The curve was from results for only 3 mice, and more experiments are therefore necessary to obtain reliable data. With respect to the survival curve, we observed a steeper decline due to the absence of early deaths among the animals used in the study. All of the survival data are still preliminary results and survival studies with larger numbers of mice are necessary. One of the characteristics of mutant p85β is that the Akt activity varies depending on the difference in growth factors. The mutant p85β gene prevented Akt activity when NGF, EGF, FGF and PDGF were used to trigger the signaling but not when insulin and IGF-1 were used to trigger the signaling. Therefore, we examined the growth factor specificity of PDGF and insulin in the cells of mice with a prolonged lifespan. By using punch biopsies from transgenic mice, we ascertained reliable protein expression of p85β transgenes in mouse dermal fibroblasts with epitope-tagged wild-type (WT) and mutant (M) p85β by immunoblotting with an anti-FLAG antibody (Fig. 5F). PDGF-induced Akt phosphorylation was examined in primary fibroblasts from a wild-type p85β transgenic mouse (mouse WT1) and a mutant p85β transgenic mouse (mouse M1). Levels of Akt phosphorylation induced by PDGF were much lower in cells with mutant p85β than in cells with wild-type p85β or in control mouse fibroblasts (Fig. 5G and Supplementary Figure 9A and 9B). Thus, mutant p85β in mouse cells prevented Akt signaling when the PDGF receptor was used to trigger the signaling, but mutant p85β in mouse cells could not prevent signaling triggered by insulin.

**Figure 6:**
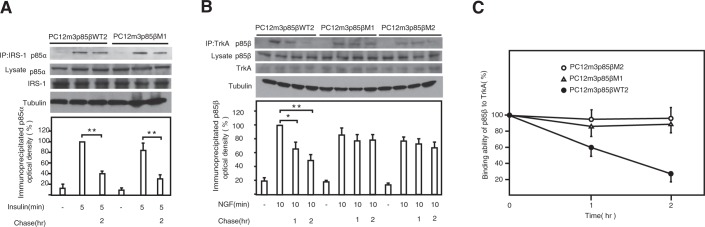
TrkA associated more stably with mutant p85β than wild-type p85β. FLAG-epitope-tagged wild-type p85β- and mutant p85β-transfected PC12m3 clone cells of PC12m3p85βWT2, PC12m3p85βM1, and PC12m3p85βM2 were serum-starved and incubated with insulin (100 ng/ml) for 5 min (**A**) or with NGF (30 ng/ml) for 10 min (**B**). Following treatment ligand was removed and the cells were incubated for 1 hour or 2 hours before lysis. Lysates were subjected to immunoprecipitation with an anti-IRS-1 antibody or anti-TrkA antibody. Binding of p85α to IRS-1 (**A**) and binding of p85β to TrkA (**B**) were detected by Western blot analysis with a p85α antibody (**A**) or anti-FLAG antibody (**B**). (**C**) Change in the binding ability of p85β to TrkA as a function of time (hours) after NGF stimulation. Data are means ± s. e. m. *P < 0.05, **P < 0.01, t-test.

NGF and insulin activate the PI3K/Akt pathway in PC12m3 cells, but NGF does not activate the pathway in PC12m321 cells. Our studies suggest that the magnitude, location, or duration of PI3K activation in response to NGF and insulin may differ. To assess these putative differences, serum-starved PC12m3, PC12m321, and wild-type or mutant p85β-transfected PC12m3 clone cells were incubated with NGF (30 ng/mL) or insulin (100 ng/mL) for 10 min, lysed, and subjected to immunoprecipitation with anti-TrkA or anti-IRS-1 antibodies. Western blotting with p85α and p85β antibodies revealed that NGF induced an association between the p85β isoform and tyrosine-phosphorylated proteins, while insulin induced an association between the p85α isoform and phosphorylated IRS-1 proteins (Fig. 6A,B). The latter association gradually decreased in both wild-type and mutant p85β-transfected PC12m3 clone cells. However, pulse-chase analysis showed that the half-life of the NGF-induced association of the p85β mutant protein did not change significantly in comparison with that of the p85β wild-type protein, and it remained constant over a period of 120 min (Fig. 6B,C). We next examined whether mutant p85β protein exhibited abnormal binding with growth factor receptors in normal human cells. Growth factor-specific receptor phosphorylation in PDGFR and insulin-R was observed in normal human fibroblasts, normal human fibroblast clone cells overexpressing p85β WT, and normal human fibroblast clone cells overexpressing p85β mutant (Supplementary Fig. 7A). On the other hand, p85β protein formed a heterodimer with the p110α catalytic subunit but not with the p110β subunit (Supplementary Fig. 7B), and p85β protein did not associate with IRS-1 protein (Supplementary Fig. 7C). Pulse-chase experiments showed that the PDGFβ-induced association of wild-type p85β protein with phosphorylated PDGFRβ protein decreased gradually from 2 to 4 hours, but the same association of mutant p85β protein with phosphorylated PDGFRβ protein did not change during the same incubation period (Supplementary Fig. 7B,D). However, the insulin-induced association of p85α protein with phosphorylated IRS-1 protein and the PDGFβ-induced association of p85α protein with phosphorylated PDGFRβ protein decreased during incubation from 2 to 4 hours in both NHDFWT2 and NHDFM1 cells (Supplementary Fig. 7C,D).

A nucleotide sequence homology search of the C-SH2 domain of p85β showed that the rat and human nucleotide sequences both consist of 321 bases and vary in 36 of those bases^15,16^ (Supplementary Fig. 8A). However, the 107 residue amino acid sequences were identical (Supplementary Fig. 8B), indicating the importance of p85β C-SH2 structural conservation across species. In contrast, a few amino acid differences were observed in the N-SH2 domain between rats and humans. Site-directed mutagenesis analysis has revealed pockets in the SH2 domain with specificity for binding the phosphotyrosines of various growth factor receptors^14,17^. Two arginines in the p85β C-SH2 domain, R623 and R641, are important for the formation of these pockets (Supplementary Fig. 2A,B). The lysine at position 660 is important for interaction with phosphotyrosine^14^ as well. This lysine in the p85β C-SH2 domain was converted to arginine by a transversion mutation in PC12m321 cells. Since arginine is the most alkaline amino acid, we hypothesize that mutant p85β has an abnormal association with NGFR and that NGF stimulation induces less PI3K/Akt activation in p85β mutant PC12m321 cells. In the case of another NGFR type receptor, PDGFR, we also assume that mutant p85β has an abnormal association with PDGFR and that PDGF stimulation induces less Akt activation in normal human fibroblast clone cells overexpressing p85β mutant.

We examined fasting blood glucose and insulin levels in transgenic mice. Fasting blood glucose levels were low only in the transgenic mice with overexpression of mutant p85β. Fasting insulin levels were high only in the same mice (Supplementary Fig. 5A,B). All of the mutant p85β transgenic mice with a prolonged lifespan were obese (Supplementary Fig. 6). We observed that the animals tended to become obese over a long period of time. We conducted additional experiments to produce 9 transgene-positive mice. The blood glucose levels and serum insulin levels in these mice are shown in Supplementary Fig. 10A,B. Like earlier observations (Fig. 4A,B), fasting blood glucose levels were low only in the transgenic mice overexpressing mutant p85β, and fasting insulin levels were high only in the same mice. Supplementary Fig. 11A shows the candidate mouse that may have a prolonged lifespan from a mutant p85β gene. A representative image of obese mice with visceral fat is shown in Supplementary Fig. 11B. Protein expression levels of FLAG-epitope-tagged mutant p85β transgenes in primary skin fibroblasts from transgenic mice are shown in Supplementary Fig. 12A. PDGF-induced Akt phosphorylation levels in primary skin fibroblasts from two mutant-type p85β transgenic mice, p85βM (−) and p85βM (+), are shown in Supplementary Fig. 12B. The results of physiological and pathological examinations of the mice used in the experiments are shown in Supplementary Tables 1 and 2. The physiological and pathological examinations showed mild underfunctioning of the liver among the obese mice that had a longer lifespan. However, in comparison with the control mice, large differences in age-related factors such as creatinine and cholesterol levels were not observed.

## Discussion

Long-lived insulin/IGF-1 signaling mutant animals such as *C. elegans, Drosophila*, and mice display enhanced basal activation of antioxidants and a concomitant reduction in oxidative damage^18,19^. We found that a disruption in NGF/PDGF signaling, but not in insulin/IGF-1 signaling, led to a prolonged lifespan. Mutant p85β gene recombinant cells exhibited strong resistance to oxidative stress compared to normal cells (Fig. 3E), and PC12m321 cells carrying a mutation in p85β showed a high level of Akt phosphorylation induced by oxidative stress (Fig. 1C). Akt increases survival by phosphorylating and inactivating Bad, which causes apoptosis^20^. We hypothesized the mechanism of resistance to oxidative stress in the mutant p85β gene as follows. Normal cells already had phosphorylated Akt because of growth factors such as EGF, FGF, and PDGF in serum and a large amount of Akt had already been used. Therefore, hardly any new PI3K/Akt activity was induced by oxidative stress. Normal cells are thus more sensitive to oxidative stress than are mutant p85β gene recombinant cells. Our results suggest that increased antioxidant protection may be associated with longevity in NGF/PDGF signaling in mutant mice.

Activation of sirtuin and FOXO also induces activation of superoxide dismutase to prevent damage induced by oxidative stress^21^. These findings indicate that the longer lifespan in mutant p85β transgenic mice may be caused by sirtuin activation and resistance to oxidative stress. FOXO transcription factors play important roles in metabolism, cellular proliferation, stress tolerance, and aging^22–24^. The growth factor-stimulated PI3K/Akt pathway negatively regulates FOXO factors by phosphorylation-mediated nuclear exclusion. The mutant p85β protein may increase the lifespan of mice by preventing negative regulation of FOXO in response to PI3K/Akt pathway inhibition. Similarly, the *Caenorhabditis elegans* longevity protein hSir2^SIRT1^ can antagonize inhibition of acetylation-induced FOXO^25^. The PI3K inhibitors LY-294002 and wortmannin slightly prolong the lifespan of *Drosophila*^26^. Target of rapamycin (TOR), which is activated by PI3K/Akt and functions in cell proliferative activity, is inhibited by rapamycin^27^, which also extends the lifespan of *Drosophila*^26^ and mice^28^. Mutant p85β acts to inhibit the PI3K/Akt pathway as effectively as rapamycin, LY-294002, and wortmannin do and it prolongs the lifespan of animals, but the molecular mechanism by which they act on the catalytic subunit of PI3K is unknown. Both p85α and p85β are mutated in several cancers^29^. These mutants show oncogenic activity; most of the p85α mutations disrupt inhibitory interactions between p85 and p110 and result in increased PI3K signaling^30–32^. Peter K. Vogt reported that p85β is a less effective inhibitor than p85α of the PI3K catalytic subunit and that this reduced level of p110 inhibition accounts for the oncogenic activity of p85β^29^. Elevated expression of p85β stimulates PI3K signaling and is linked to tumor progression^33^. p85β is mutated in endometrial cancer and the mutation activates PI3K signaling^32^. p85β mutation or p85β activation is thought to be involved in tumor progression by promoting cell proliferation. In our study, we found that a lysine to arginine mutation of p85β works to inhibit PI3K and also to suppress proliferation of rat adrenal medulla pheochromocytoma cells (PC12 cells). No cancer was found in mice expressing the mutant p85β, suggesting that mutant p85β may suppress the carcinogenesis of cells. However, this experiment requires more detailed analysis. A previous study by Jie Xu^34^ has showed that life extension of IGF1R+/− mice is dependent on the genetic background of the mice and that C57BL/6J mice and 129/SyPas mice will yield different results. In the present study, we showed that p85β mutation results in decreased activation of PDGFR and NGFR, which in turn increases longevity. Additional research is required in the future to verify similar results in 129/SyPas mice. One of the limitations of the present study is that there is no evidence for alterations in NGF or PDGF signaling in tissues in the mice. In the future, additional research is required to study NGF and PDGF signaling in mouse tissues.

## Materials and Methods

### Cells and cell culture

PC12 cells^35^ were cloned from a rat pheochromocytoma by L. A. Green in 1976 and were kindry provided by Dr. K. Fushimi (Okayama Medical School). PC12m3 and PC12m321 mutant cell lines used here were derived from PC12 cells. Owing to the widespread use of PC12 cells in various culture conditions, spontaneous variants are often encountered. We obtained a variant cell line, PC12m321, from PC12 parental cells in an acidic culture condition. When PC12 parental cells were cultured for 2 weeks under an acidic condition of Cl^−^, several clones survived. By using the ring isolation procedure, ten colonies were selected and propagated in mass culture. They were termed PC12m1, PC12m2, PC12m3, and so on. PC12m3 cells were divided into several cultures, and individual cultures were subcultured separately. These were termed PC12m31, PC12m32, PC12m33, and so on. Among these cultures, PC12m32 cells showed a high rate of induction of neurite outgrowth caused by osmotic shock^36^. PC12m321 is a sister culture of PC12m32 cells. PC12 parental cells, PC12m3, and PC12m321 cells were cultured in Dulbecco’s modified Eagle’s medium (DMEM) supplemented with 0.35% glucose, 10% horse serum, 5% fetal bovine serum (FBS), and 100 units/ml kanamycin. Mouse diploid fibroblasts were obtained from control mice and transgenic mice. By using punch biopsies from transgenic mice, the Akt activity of mouse dermal fibroblasts with FLAG epitope-tagged wild-type (WT) or mutant (M) p85β was examined.

NHDF is a normal human diploid fibroblast strain derived from newborn male foreskin. This cell strain was obtained from TOYOBO Life Science (Osaka Japan) and used for transfection of p85β DNA. All mouse and human fibroblasts were maintained in Eagle’s minimal essential medium (MEM) supplemented with 10% fetal bovine serum and 60 μg/ml kanamycin. All cells were grown in culture flasks without poly-D-lysine coating at 37 °C in 5% CO_2_.

### Plasmid construction

PCR products of p85α and p85β isolated from PC12, PC12m3, and PC12m321 cells were subcloned into the EcoRI site of pCRR-XL-TOPOR using a TOPORXLPCR cloning kit (Life Technologies Corporation, Grand Island, NY, USA). Full-length p85α, wild-type p85β, and mutant p85β were directly subcloned into the EcoRI site of the mammalian expression vector pcDNA3.1(−) for transfection into PC12 cells or rat fibroblasts. To construct FLAG-tagged alleles of wild-type or mutant p85β, pcDNA3.1(−) plasmid DNA with wild-type p85β or mutant p85β was subcloned into plain pcDNA3.1(−) by PCR with primers containing a FLAG-epitope coding sequence using KOD plus polymerase (Toyobo, Osaka, Japan), generating kozak-ATG-FLAG-PI3k p85β in the pcDNA3.1(−) plasmid. PCR amplification was carried out with forward primer 5′-AAGAATT**CACC**ATGGACTACAAGGACGACGATGACAAGATGGCAGGTGCTGAAGGC-3′ and reverse primer 5′-ATAAGCTTCAGCGTGCTGCAGGGGG-3′. The forward primer includes a Kozak sequence (in bold) followed by the ATG start codon (underlined) and the sequence encoding the FLAG epitope (double underlined). The complete sequence was verified by DNA sequencing.

### Primary culture

Mouse dermal diploid fibroblasts were obtained using the punch biopsy procedure on control and p85β-transgenic mice. Tissues from the skin of young adult mice were divided into small portions (1 to 3 mm^3^). Twenty pieces of tissue were anchored to 100-mm dishes and incubated in Eagle’s minimal essential medium containing 10% fetal bovine serum, 60 mg/L kanamycin, and 40 nmol/L glutamine. Once fibroblast growth had started from the explant, the number of growth starting positions was counted under a microscope. When one cell divides 10 times, the number of cells becomes one thousand (10 population doublings (PDs)). When about twenty cells had become 200,000–400,000 cells (12–14 PDs), they were harvested for Western blot analysis. The mouse diploid fibroblasts were used for examination of Akt phosphorylation induced by PDGF and insulin before senescence or the appearance of spontaneously immortalized cells. The mouse colony was proven to be specific pathogen free throughout the period of experimentation. Testing for the presence of mouse hepatitis virus, *Mycoplasma pulmonis*, and *Clostridium piliforme* yielded negative results. Like the insulin and blood glucose investigations blood samples were drawn from the mice for physiological and pathological examinations. The serum collected from the blood sample was used for investigation of several parameters.

### Transgene construction and mutant p85β transgenic animal production

Mutant p85β DNA was inserted via the EcoRI site into the mammalian expression vector pcDNA3.1(−). The constructed plasmid consisted of the CMV promoter, mutant p85β cDNA, BGH poly(A) signal, SV40 promoter, neo-resistance gene, and SV40 poly(A) signal, in that order (Supplementary Fig. 3). The mutant p85β transgene was excised from its plasmid by SalI and SfoI digestion and purified by gel electrophoresis and ultracentrifugation. The purified mutant p85β transgene was microinjected into fertilized C57BL/6 mouse eggs (Unitech, Inc., Chiba, Japan). The microinjected eggs obtained this way were returned to the uterus of a female mouse, which led to the birth of 8 F0 mice (3 females, 8 males). Of these, one female and one male tested positive for mutant p85β DNA by PCR, while the other six tested negative. In the second experiment, microinjections were performed in the same manner, leading to the birth of 5 F0 mice (3 females and 2 males). Of these, only 1 male was positive for mutant p85β DNA whereas the others tested negative. Potential founders were analyzed for the presence of the transgene by polymerase chain reaction of mouse genomic DNA isolated from ear punch specimen using a CMV-specific primer and rat p85β primer: GT-CMV-F2, 5′-ATGACCTTATGGGACTTTCCTACTT-3′ (552–576) as the forward primer and KBK71- p85β-R2, 5′-TCAGTGAATACTGGTCCTCAGTCTC-3′ (2963–2939) as the reverse primer. Animal care and experimentation were performed according to the study guidelines established by the Kibi International University Subcommittee on laboratory animal care, handling and termination. The study protocol was approved by the Kibi International University Subcommittee on laboratory animal care, handling and termination (Approval number A17-03). The details regarding the mice used in the study are shown in Table 1. The details of the experimental mice used in the second set of experiments are provided in Table 2. Organ weight in grams of the mice used in the experiment are shown in Table 3.

**Table 1:** Details of the mice used in the study.

Sources	Generation	No.	Birth	Death	Life Span	Sex	Experiments
Wild-type	F0	1	5/6/2009	2/25/2011	688	♂	
Wild-type	F0	2	5/6/2009	4/14/2011	708	♀	
Wild-type	F0	3	4/8/2009	5/7/2011	759	♀	
Wild-type	F0	4	4/8/2009	5/14/2011	766	♀	
Wild-type	F0	5	4/8/2009	5/23/2011	775	♂	
Wild-type	F0	6	4/8/2009	6/14/2011	797	♂	
Wild-type	F0	7	4/8/2009	6/29/2011	812	♂	
Wild-type	F0	8	11/5/2014	2/23/2017	841	♂	INC GLU Akt
Wild-type	F0	9	11/5/2014	3/4/2017	851	♂	INC GLU Akt
Wild-type	F0	10	5/6/2009	9/10/2011	857	♂	
Wild-type	F0	11	11/5/2014	4/6/2017	884	♂	INC GLU Akt
P85bWT	F0	1	11/5/2014	12/19/2016	775	♀	INC GLU Akt
P85bWT	F0	2	11/5/2014	1/5/2017	792	♀	INC GLU Akt
P85bWT	F0	3	11/5/2014	2/23/2017	841	♀	INC GLU Akt
P85bM-tg(−)	F1	1	3/31/2010	2/7/2012	677	♀	
P85bM-tg(−)	F1	2	3/31/2010	2/9/2012	679	♀	
P85bM-tg(−)	F1	3	3/31/2010	2/23/2012	693	♀	
P85bM-tg(−)	F1	4	3/31/2010	3/3/2012	702	♀	
P85bM-tg(−)	F1	5	3/31/2010	4/2/2012	732	♂	
P85bM-tg(−)	F1	6	3/31/2010	4/18/2012	748	♂	
P85bM-tg(−)	F1	7	3/31/2010	4/19/2012	749	♂	
P85bM-tg(−)	F1	8	3/31/2010	4/29/2012	759	♂	
P85bM-tg(−)	F1	9	3/31/2010	5/15/2012	775	♂	
P85bM-tg(−)	F1	10	3/31/2010	5/16/2012	776	♂	
P85bM-tg(−)	F1	11	3/31/2010	6/1/2012	792	♂	
P85bM-tg(−)	F1	12	3/31/2010	6/1/2012	792	♂	
P85bM-tg(−)	F1	13	3/31/2010	3/3/2012	794	♂	
P85bM-tg(−)	F1	14	3/31/2010	6/4/2012	795	♀	
P85bM-tg(−)	F1	15	3/31/2010	6/5/2012	796	♂	
P85bM-tg(−)	F1	16	3/31/2010	6/20/2012	811	♂	
P85bM-tg(−)	F1	17	3/31/2010	7/6/2012	827	♀	
P85bM-tg(+)	F1	1	3/31/2010	10/1/2012	914	♂	
P85bM-tg(+)	F1	2	3/31/2010	10/5/2012	918	♀	
P85bM-tg(+)	F1	3	3/31/2010	10/26/2012	939	♂	
P85bM-tg(+)	F1	4	3/31/2010	10/26/2012	939	♂	
P85bM-tg(+)	F1	5	3/31/2010	11/5/2012	949	♂	
P85bM-tg(+)	F1	6	3/31/2010	12/4/2012	978	♀	
P85bM-tg(+)	F2	1	8/31/2013	2/14/2016	897	♂	
P85bM-tg(+)	F2	2	8/31/2013	3/22/2016	934	♀	
P85bM-tg(+)	F2	3	8/31/2013	3/28/2016	940	♀	
P85bM-tg(+)	F2	4	8/31/2013	4/18/2016	961	♂	INC GLU Akt
P85bM-tg(+)	F2	5	8/31/2013	4/23/2016	966	♀	INC GLU Akt
P85bM-tg(+)	F2	6	8/31/2013	5/4/2016	977	♂	INC GLU Akt

**Table 2:** Details of mice used for the second set of experiments.

Sources	Generation	No.	Birth	Weight (g)	Sex	Experiments
Wild-type	—	1	2/7/2018	21.2	♂	INS GLU Akt OW Phy Path
Wild-type	—	2	2/7/2018	—	♂	
Wild-type	—	3	2/7/2018	—	♂	
Wild-type	F2	1	11/28/2017	—	♀	INS GLU Phy Path
Wild-type	F2	2	11/28/2017	—	♀	INS GLU
Wild-type	F2	3	11/28/2017	—	♀	INS GLU
P85bM	F0	1	6/7/2017	28.2	♀	INS GLU
P85bM	F1	1	8/2/2017	32.3	♂	INS GLU
P85bM	F1	2	8/2/2017	24.8	♀	INS GLU Akt
P85bM	F1	3	8/22/2017	31.2	♂	INS GLU OW Phy Path
P85bM	F1	4*	9/14/2017	34.0	♂	INS GLU Phy Path
P85bM	F1	5*	9/14/2017	44.4	♂	INS GLU OW Phy Path
P85bM	F1	6	9/14/2017	26.6	♀	INS GLU
P85bM	F2	1*	11/28/2017	25.4	♀	INS GLU Akt Phy Path
P85bM	F2	2	11/28/2017	25.5	♀	INS GLU

INS: fasting insulin level. GLU: fasting glucose level. Akt: Akt phosphorylation assay. OW: organ weight. Phy: physiological examination. Path: pathological examination.

* indicates candidate mice (p85βM(+)) that may have a prolonged lifespan from a mutant p85β gene.

**Table 3:** Organ weight (grams) of mice used for experiments.

Sources	Age (days)	Sex	Weight (g)	Heart	Liver	Pancreas	Spleen	Kidney	Thymus
Wild-type (No. 1 of Wild-type in Table 2)	132	♂	29.1	0.14	1.31	0.25	0.07	017, 0.21	0.06
P85bM (No. 3 of P85bM F1 in Table 2)	301	♂	29.8	0.14	1.39	0.24	0.08	0.19, 0.18	0.04
P85bM (No. 5* of P85bM F1 in Table 2)	278	♂	47.0	0.17	2.07	0.24	0.10	0.27, 0.25	0.11

### Stable transfections

To obtain permanent clone cells overexpressing p85β WT or p85β mutant, plasmid pcDNA3.1 (−)-constructed p85β was transfected into PC12 cells, rat immortal fibroblasts, and human primary fibroblasts. At approximately 90% confluence in 100-mm dishes, cultured PC12m3 cells and normal human diploid fibroblasts were transfected with 20 μg supercoiled mutant p85β or wild-type p85β DNA by calcium phosphate/DNA coprecipitation^37^. After 7 hours of incubation, the cells were washed and cultured in fresh medium. Seventeen hours later, the cells were subcultured at a 1:4 dilution. The transfected cells were selected by exposure to medium containing 400 μg/ml G418. The selection medium was changed every 3 days. Using the ring isolation procedure, 10 to 20 G418-resistant colonies were selected from mutant p85β or wild-type p85β DNA-transfected PC12m3 cells, rat immortal fibroblasts (RHF), and normal human diploid fibroblasts (NHDF) and propagated in a mass culture. These were termed PC12m3M1, PC12m3M2…, PC12m3WT1, PC12m3WT2…, NHDFM1, NHDFM2…, NHDFWT1, NHDFWT2, NHDFWT3…, and so on. These PC12m3 and normal human diploid fibroblast strains were examined for sensitivity to NGF, EGF, FGF, insulin, IGF-1, serum, PDGF, and H_2_O_2_.

### Growth curve

Exponentially growing PC12 cells were plated in eight 25 cm^2^ culture flasks at a density of 1 × 10^5^ cells/flask. The cells were then incubated for eight days. Cell number and viability were determined by trypan blue dye exclusion analysis every 8 days. The PC12 cells were subjected to three experiments and statistical processing was performed.

### Western blot analysis

Akt, ERK, and FOXO activities were determined as described previously^38^. Briefly, PC12 parental and PC12 mutant cells were plated at a density of 1 × 10^6^ cells/25 cm^2^ in a flask of serum-containing medium and cultured for 3 days. Then the culture medium was replaced with medium containing 0.5% FBS, and the cells were cultured for a further 48 h. PC12 parental, PC12m3 and PC12m321 cells were then treated for 10 or 30 min with NGF (30 ng/ml) or exposed to heat shock (44 °C) for 30 min. Akt, ERK, and FOXO activities in cell lysates were then assayed. The cells were lysed in a lysing buffer. Aliquots of the lysates (10–15 μg) from each sample were fractionated on an SDS-10% polyacrylamide gel and transferred to polyvinylidene difluoride membranes. The blots were probed with antibodies specific for phospho-Akt, phospho-ERK1/2, phospho-FOXO, total Akt, or total FOXO (New England BioLabs; Beverly, MA) at a dilution of 1:1000 in blocking buffer (5% nonfat dry milk in PBS) for 12 h at 4 °C. The blots were probed with a secondary antibody, horseradish peroxidase-linked anti-rabbit IgG, at a dilution of 1:2000 in blocking buffer for 60 min at 25 °C. The blots were stained for 1 min using a nucleic acid chemiluminescence reagent (LumiGLO chemiluminescent reagent, Kirkegaard and Perry Laboratories, Gaithersburg, MD, USA) and exposed to x-ray film.

### Immunoprecipitation

After starvation, cells were treated with insulin or NGF for 10 min and then lysed in Nonidet P-40 lysis buffer as described in ref.^4^. The lysates were subjected to immunoprecipitation with an anti-IRS-1 antibody or anti-TrkA antibody and immobilized on G-Sepharose beads. The lysates or the precipitates were subjected to immunoblotting and visualized by an enhanced chemiluminescence system (LumiGLO chemiluminescent reagent, Kirkegaard and Perry Laboratories, Gaithersburg, MD, USA).

### Pulse-chase assay

PC12 parental and PC12 mutant cells were plated at a density of 1 × 10^6^ cells/25 cm^2^ in a flask of serum-containing medium and cultured for 3 days. Normal human diploid fibroblasts (NHDF) transfected with wild-type p85β DNA (NHDFWT-1, NHDFWT-2) and mutant p85β DNA (NHDFM-1) were plated at a density of 3 × 10^5^ cells/25 cm^2^ in a flask of serum-containing medium and cultured for 3 days. Then the culture medium was replaced with 0.5% FBS-containing medium, and the cells were cultured for a further 48 hours. PC12 parental, PC12m3 and PC12m321 cells were then treated for 5 min with insulin (100 ng/ml) or for 10 min with NGF (30 ng/ml). NHDFWT-1, NHDFWT-2 and NHDFM-1 cells were treated for 5 min with insulin (100 ng/ml) or for 10 min with PDGF (8 ng/ml). The medium was aspirated from cultures, and the cells were washed twice with FBS-free medium, and then fresh 0.5% FBS-containing medium was added. The cells were incubated for 0, 1, 2, or 4 hours and then lysed, and the cell lysates were subjected to immunoprecipitation and Western blot analysis.

### Survival

Single-cell suspensions of PC12m3, PC12m321, and mutant p85β-transfected PC12m3 cells were obtained by trituration in DMEM. To examine the toxicity of oxidative stress, PC12m3 and PC12m321 cells were treated with H_2_O_2_ for 10 min at various concentrations ranging from 0.1 to 30 mM or for 120 min at various concentrations ranging from 0.05 to 0.3 mM in DMEM containing 10% horse serum using a 1.5-ml microcentrifuge tube at 37 °C. Then the cells were centrifuged at 1700 rpm for 2 min and the supernatant was aspirated to remove the H_2_O_2_. The precipitated cells were washed three times by adding 1 ml DMEM followed by centrifugation and aspiration. After washing, cells were plated in 25-cm^2^ flasks and incubated for 7–10 days prior to colony staining, and then the colonies were counted. Each value is the mean ± S. D. for cells sampled from three independent experiments.

### Reverse-Transcription PCR

Total RNA was isolated from PC12, PC12m3 and PC12m321 cells with TRIzol reagent (Invitrogen, Carlsbad, CA) according to the manufacturer’s protocol. Briefly, 1 μg of total cellular RNA was reverse-transcribed by a First-Strand cDNA Synthesis Kit (Amersham, Buckinghamshire, UK). Primers used for amplification of p85α were 5′-ATGAGCGCAGAGGGGTACCA-3′ as the forward primer and 5′-TCATCGCCTCTGTTGTGCATAT-3′ as the reverse primer for amplifying a 2179-bp fragment. Primers used for amplification of p85β were the forward primer 5′-GACTAAATGGTGGACTCTGTGA-3′ and the reverse primer 5′-AGACAGACATGGACAGGGAGGC-3′ for amplifying a 2401-bp fragment. The following conditions were used for PCR: 30 cycles of 94 °C for 30 s, 55 °C for 30 s, 72 °C for 145 s, followed by 72 °C for 7 min for final extension. The PCR products were separated on a 1% agarose gel, visualized under UV light, and photographed. The results were analyzed by Quantity One 4.6.2 software for optical density.

### Nucleotide sequence analysis

Mutation of the p85α and p85β genes of PC12 parental, PC12m3 and PC12m321 cells was detected by sequencing after construction of cDNA plasmid pCRR-XL-TOPOP. DNA sequence determination was carried out by the dideoxy chain determination method^39^.

### Statistical analysis

All data are shown as means ± standard deviation (SD). Statistical significance was determined by analysis of variance (ANOVA) using the SPSS 12.0 software package or unpaired t-test. The level for statistical differences was set at P < 0.05 or 0.01.

## Supplementary information

Dataset 1

## Data Availability

The data generated in the study is present in the manuscript and its Supplementary Files.

## References

[1] Ahn JY, Rong R, Liu X, Ye K (2004). PIKE/nuclear PI 3-kinase signaling mediates the antiapoptotic actions of NGF in the nucleus. EMBO J..

[2] Hooshman D-RR (2000). The PI3-kinase isoforms p110α and p110β have differential roles in PDGF- and insulin-mediated signaling. J. Cell Science.

[3] Posern G, Saffrich R, Ansorge W, Feller SM (2000). Rapid lamellipodia formation in nerve growth factor-stimulated PC12 cells is dependent on Rac and PI3K activity. J. Cell Physiol..

[4] Gami MS, Iser WB, Hanselman KB, Wolkow CA (2006). Activated AKT/PKB signaling in *C. elegans* uncouples temporally distinct outputs of DAF-2/insulin-like signaling. BMC Dev. Biol..

[5] Kloet DE, Burgering BM (2011). The PKB/FOXO switch in aging and cancer. Biochim. Biophys. Acta..

[6] Sedding DG (2008). FoxO transcription factors in oxidative stress response and aging–a new fork on the way to longevity?. Biol. Chem..

[7] Anderson DH (2010). p85 plays a critical role in controlling flux through the PI3K/PTEN signaling axis through dual regulation of both p110 (PI3K) and PTEN. Cell Cycle.

[8] Farias EF, Marzan C, Mira-y-Lopez R (2005). Cellular retinol-binding protein-I inhibits PI3K/Akt signaling through a retinoic acid receptor-dependent mechanism that regulates p85-p110 heterodimerization. Oncogene.

[9] Waksman G (1993). Binding of a high affinity phosphotyrosyl peptide to the Src SH2 domain: crystal structures of the complexed and peptide-free forms. Cell.

[10] Suenaga A (2005). Novel mechanism of interaction of p85 subunit of phosphatidylinositol 3-kinase and ErbB3 receptor-derived phosphotyrosyl peptides. J. Biol. Chem..

[11] Myers (1992). IRS-1 activates phosphatidylinositol 3-kinase by associating with src homology 2 domains of p85. Proc. Natl. Acad. Sci. USA.

[12] Waugh C (2009). Phosphoinositide (3,4,5)-triphosphate binding to phosphoinositide-dependent kinase 1 regulates a protein kinase B/Akt signaling threshold that dictates T-cell migration, not proliferation. Mol. Cell Biol..

[13] Kano Y (2004). Heat shock induces neurite outgrowth in PC12m3 cells via the p38 mitogen-activated protein kinase pathway. Brain Res..

[14] Yoakim M (1994). Genetic analysis of a phosphatidylinositol 3-kinase SH2 domain reveals determinants of specificity. Mol. Cell. Biol..

[15] Carpenter CL (1990). Purification and characterization of phosphoinositide 3-kinase from rat liver. J. Biol. Chem..

[16] Kumar A (2011). Nuclear but not cytosolic phosphoinositide 3-kinase beta has an essential function in cell survival. Mol. Cell Biol..

[17] McGlade CJ (1992). SH2 domains of the p85 alpha subunit of phosphatidylinositol 3-kinase regulate binding to growth factor receptors. Mol. Cell Biol..

[18] Vanfleteren JR (1993). Oxidative stress and aging in. Caenorhabditis elegans. Biochem. J..

[19] Clancy DJ (2001). Extension of lifespan by loss of CHICO, a *Drosophila* insulin receptor substrate protein. Science.

[20] Datta SR (1997). Akt phosphorylation of BAD couples survival signals to the cell-intrinsic death machinery. Cell.

[21] Akasaki Y (2014). FOXO transcription factors support oxidative stress resistance in human chondrocytes. Arthritis Rheumatol..

[22] Accili D, Arden KC (2004). FoxOs at the crossroads of cellular metabolism, differentiation, and transformation. Cell.

[23] Van der Horst A, Burgering BM (2007). Stressing the role of FoxO proteins in lifespan and disease. Nat. Rev. Mol. Cell Biol..

[24] Sandri M (2012). FOXOphagy path to inducing stress resistance and cell survival. Nat. Cell Biol..

[25] Van der Horst A (2004). FOXO4 is acetylated upon peroxide stress and deacetylated by the longevity protein hSir2*SIRT1*. J. Biol. Chem..

[26] Moskalev AA, Shaposhnikov MV (2010). Pharmacological inhibition of phosphoinositide 3 and TOR kinases improves survival of *Drosophila melanogaster*. Rejuvenation Res..

[27] Ayral-Kaloustian S (2010). Hybrid inhibitors of phosphatidylinositol 3-kinase (PI3K) and the mammalian target of rapamycin (mTOR): design, synthesis, and superior antitumor activity of novel wortmannin-rapamycin conjugates. J. Med. Chem..

[28] Harrison DE (2009). Rapamycin fed late in life extends lifespan in genetically heterogeneous mice. Nature.

[29] Ito Y, Hart JR, Ueno L, Vogt PK (2014). Oncogenic activity of the regulatory subunit p85β of phosphatidylinositol 3-kinase (PI3K). Proc. Natl. Acad. Sci. USA.

[30] Wu H (2009). Regulation of Class IA PI3-kinases: C2 domain contacts inhibit p85/p110alpha and are disrupted in oncogenic p85 mutants. Proc. Natl. Acad. Sci. USA.

[31] Sun M, Hillmann P, Hofmann BT, Hart JR, Vogt PK (2010). Cancer-derived mutations in the regulatory subunit p85alpha of phosphatidylinositol 3-kinase function through the catalytic subunit p110alpha. Proc. Natl. Acad. Sci. USA.

[32] Cheung LW (2011). High frequency of PI3KR1 and PI3KR2 mutations in endometrial cancer elucidates a novel mechanism for regulation of PTEN protein stability. Cancer Discov..

[33] Cortes. I (2012). p85βphosphatidylinositol 3-kinase subunit regulates tumor progression. Proc. Natl. Acad. Sci. USA.

[34] Xu (2014). Longevity effect of IGF-1R(+/−) mutation depends on genetic background-specific receptor activation. Aging Cell..

[35] Greene LA, Tischler AS (1976). Establishment of a noradrenergic clonal line of rat adrenal pheochromocytoma cells which respond to nerve growth factor. Proc. Natl. Acad. Sci. USA.

[36] Kano Y (2007). Osmotic shock-induced neurite extension via activation of p38 mitogen-activated protein kinase and CREB. Brain Research.

[37] Gorman CM, Padmanabhan R, Howard BH (1983). High efficiency DNA-mediated transformation of primate cells. Science.

[38] Ueki K (2003). Positive and negative roles of p85α and p85β regulatory subunits of phosphoinositide 3-kinase in insulin signaling. J. Biol. Chem..

[39] Sanger F, Nicklen S, Coulson AR (1977). DNA sequencing with chain-terminating inhibitors. Proc. Natl. Acad. Sci. USA.

