# Supplementary material for: C-SH2 point mutation converts p85β regulatory subunit of phosphoinositide 3-kinase to an anti-aging gene
Source: Sci Rep. 2019 Sep 3;9:12683. doi: 10.1038/s41598-019-48157-6 (PMC6722097; doi:10.1038/s41598-019-48157-6)
Supplement: Supplementary file 1 — Dataset 1 [file 41598_2019_48157_MOESM1_ESM.docx]

**C-SH2 point mutation converts p85β regulatory subunit of phosphoinositide 3-kinase to an anti-aging gene**

Yoshio Kano^1*^, Fukumi Hiragami^1^, Hirotoshi Motoda^1^, Junichi Akiyama^1^, Yoshihisa Koike^2,3^, Yutaka Gomita^2,4^, Shigeki Inoue^1^, Akihiko Kawaura^1^, Tomohisa Furuta^2^ and Kenji Kawamura^1^

**^1^**Graduate School of Health Science, Kibi International University, 8-Iga-machi, Takahashi, Okayama, 716-8508, Japan

**^2^**Research Institute of Health and Welfare, Kibi International University, 8-Iga-machi, Takahashi, Okayama, 716-8508, Japan

**^3^**Department of Occupational Therapy, Faculty of Health and Welfare, Prefectural University of Hiroshima, Mihara City, Hiroshima 723-005, Japan

**^4^**Department of Pharmacy, Okayama University Hospital Pharmacy, 2-5-1 Shikata-cho, Okayama 700-8558, Japan

*To whom correspondence should be addressed: E-mail: yoshio@kiui.ac.jp

**Supplementary information**

**Supplementary table 1**. Physiological analysis of mice used for experiments.

| Test item | Standard Value | Wild-type 1 ♂ | Wild-type F2-1 ♀ | P85bM(-) F1- 3 ♂ | P85bM(+) F1- 4* ♂ | P85bM(+) F1- 5* ♂ | P85bM(+) F2-1*♀ |
| --- | --- | --- | --- | --- | --- | --- | --- |
| TP (g/dL) | 4.7-5.7 | 4.6 | 5.0 | 4.8 | 4.6 | 5.2 | 4.4 |
| ALB (g/dL) | 2.8-3.5 | 3.2 | 3.4 | 3.2 | 3.2 | 3.4 | 3.2 |
| Na (mEq/L) | 148-173 | 158 | 155 | 162 | 156 | 154 | 158 |
| K (mEq/L) | 4.0-7.6 | 4.8 | 4.2 | 5.2 | 6.0 | 4.4 | 4.4 |
| Cl (mg/L) | 103-120 | 100 | 106 | 110 | 98 | 103 | 98 |
| Ca (mg/L) | 9.6-12.5 | 9.0 | 9.4 | 9.3 | 9.4 | 8.9 | 8.8 |
| LDH (IU/L) | 345-1290 | 626 | 506 | 709 | 712 | 424 | 488 |
| LAP (IU/L) | 48-66 | 26 | 32 | 31 | 32 | 39 | 32 |
| AMY (IU/L) | 2190-4090 | 2048 | 1586 | 2599 | 2586 | 3233 | 1722 |
| T-BIL (mg/dL) | 0.04-0.10 | 0.10 | 0.07 | 0.08 | 0.06 | 0.07 | 0.10 |
| T-KB (mmol/L) | 66-1148 | 328 | 631 | 247 | 128 | 307 | 232 |

Physiological examination of the 6 mice that are represented in table 2.

(* ) indicates candidate mice (p85bM(+)) that may have a prolonged lifespan due to the mutant p85 b gene.

**Supplementary table 2**. Pathological analysis of mice used for experiments.

| Test item | Standard Value | Wild-type 1 ♂ | Wild-type F2-1 ♀ | P85βM(-) F1- 3 ♂ | P85βM(+) F1- 4* ♂ | P85βM(+) F1- 5* ♂ | P85βM(+) F2-1*♀ |
| --- | --- | --- | --- | --- | --- | --- | --- |
| BUN (mg/dL) | 16.2-26.4 | 25.8 | 17.7 | 40.1 | 27.8 | 22.3 | 27.4 |
| CRE (mg/dL) | 0.08-0.12 | 0.12 | 0.12 | 0.13 | 0.12 | 0.10 | 0.14 |
| IP (mg/dL) | 8.5-12.0 | 7.8 | 9.2 | 8.8 | 8.6 | 6.6 | 7.8 |
| AST (IU/L) | 47-108 | 104 | 94 | 72 | 88 | 123 | 114 |
| ALT (IU/L) | 16-38 | 24 | 28 | 24 | 40 | 51 | 20 |
| ALP (IU/L) | 300-700 | 272 | 328 | 296 | 268 | 275 | 312 |
| T-CHO (mg/dL) | 93-200 | 78 | 76 | 68 | 90 | 78 | 56 |
| F-CHO (mg/dL) | 12-38 | 20 | 21 | 18 | 18 | 17 | 14 |
| E-CHO (mg/dL) | 77-165 | 58 | 55 | 50 | 72 | 61 | 42 |
| LDL-C (mg/dL) | 3-14 | 2 | 10 | 5 | 8 | 6 | 6 |
| HDL-C (mg/dL) | 45-107 | 50 | 39 | 43 | 56 | 44 | 32 |

Pathological examination of the 6 mice that are represented in table2.

(* ) indicates candidate mice (p85β M(+)) that may have a prolonged lifespan due to the mutant p85β gene.

**Supplementary Fig. 1**


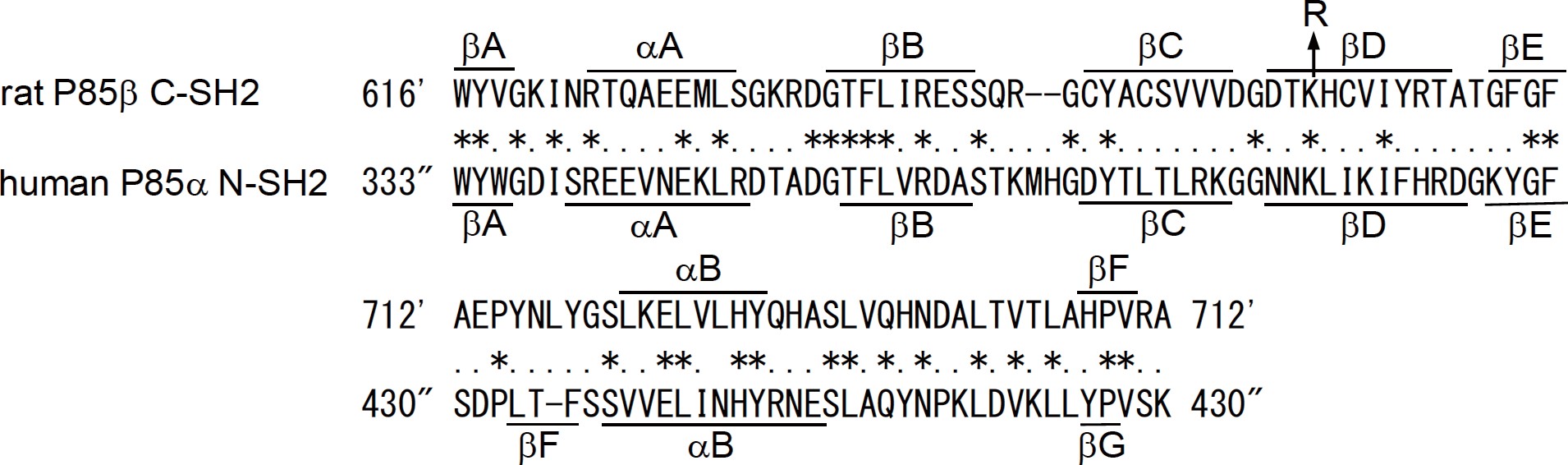
**A B**


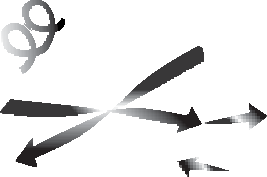

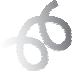

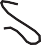

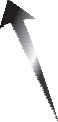

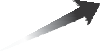

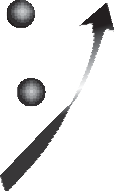

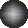

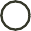

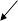


（R-623)

（R-641)

P

（K-660 → R660)

Structural model for the C-SH2 domain in the mutant p85β gene of PC12m321 cells.

(**A**) Comparison of the sequences of rat p85 C-SH2 and human p85 N-SH2 constructs^17^. (**B**) Structural model for the p85β C-SH2 domain. indicates substitution of arginine (R) for lysine (K) at position 660 of p85β in PC12m321 cells.

1. **Supplementary Fig. 2**


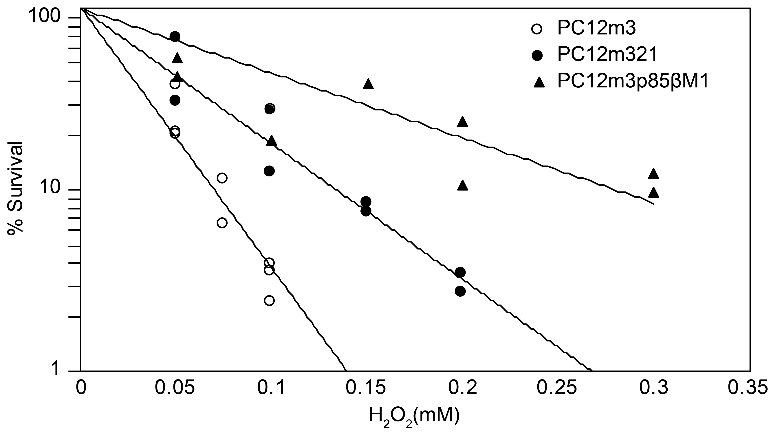


Effects of oxidative stress on the survival of PC12m3 and PC12m321 cells. PC12m3 (○), PC12m321 (●) and a mutant p85β-transfected PC12m3 cell clone (∆) cells were treated with H_2_O_2_ for 120 min at various concentrations ranging from 0.05 mM to 0.3 mM.

**Supplementary Fig. 3**

**

**

**

Growth curve of PC12 cells grown in 25 cm^2^ culture flasks. Each time point represents the mean of three flasks. The PC12 cells were shown to have an S-shaped growth curve by analysis using GraphPad Prism8 software. (A) Growth curve of PC12 parental and PC12m321 cells. (B) Growth curve of mutant p85β-expressing PC12m3 cells (PC12m3p85βM1 and M2) and wild-type p85β-expressing PC12m3 cells (PC12m3 p85βWT1 and WT2). **P<0.01, t-test.

**Supplementary Fig. 4**


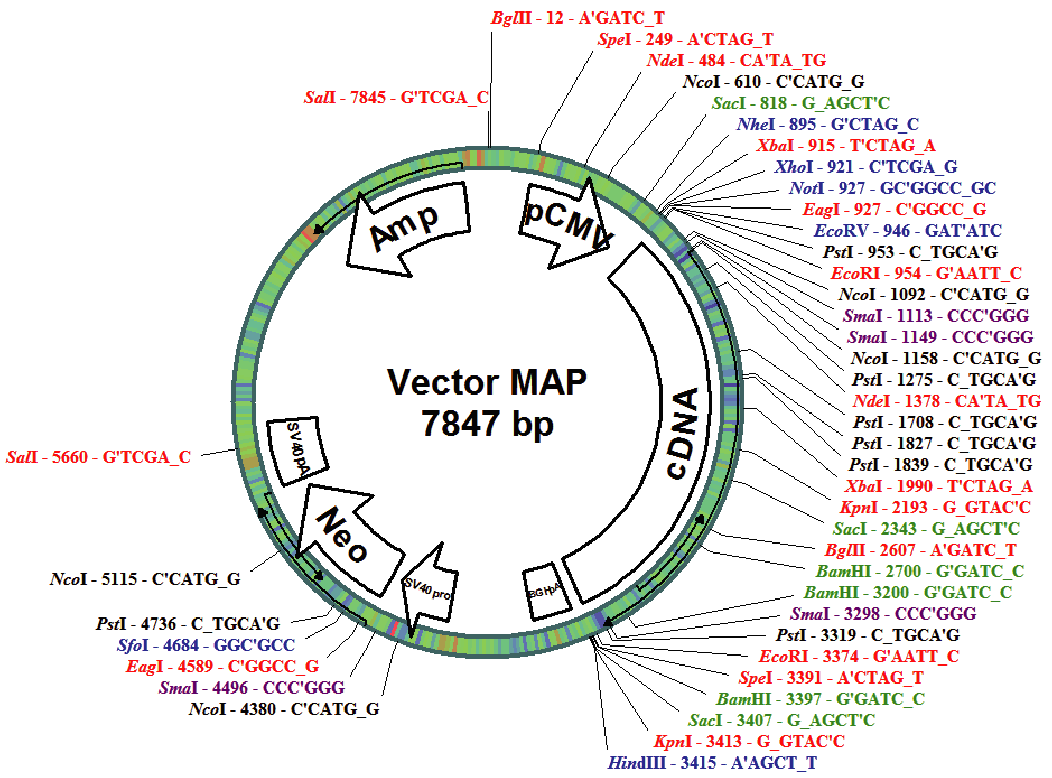


Construction of a mutant p85β transgene. Mutant p85β DNA was inserted through the EcoRI site into the mammalian expression vector pcDNA3.1(-). The constructed plasmid consisted of the CMV promoter, mutant p85β cDNA, BGH poly(A) signal, SV40 promoter, neo-resistance gene and SV40 poly(A) signal, in that order. The mutant p85β transgene was excised from its plasmid by SalI and SfoI digestion and microinjected into fertilized C57BL/6 mouse eggs.

**Supplementary Fig. 5**


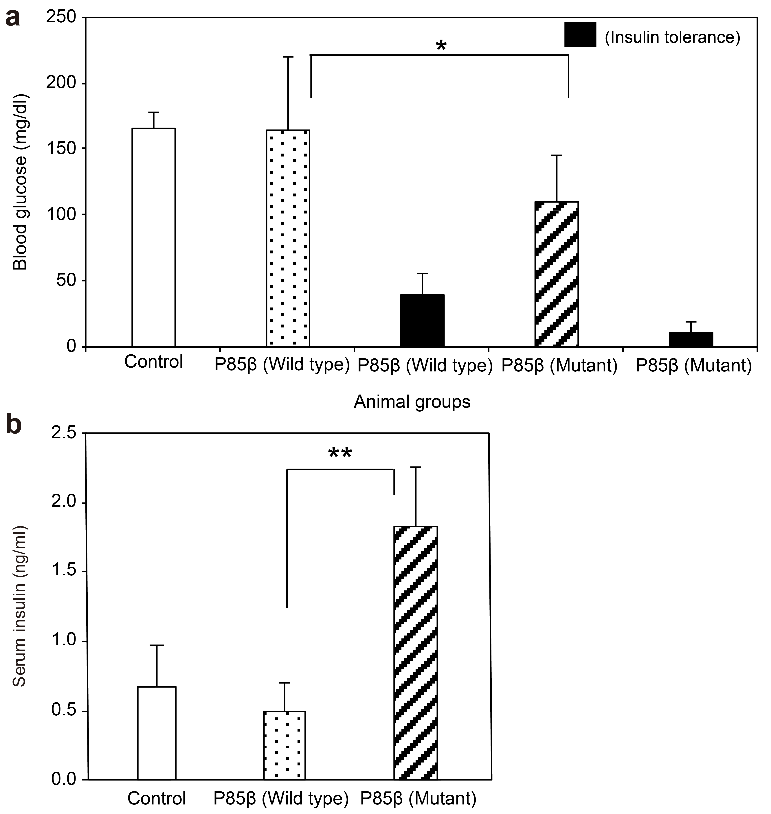


Fasting blood glucose and insulin levels in transgenic mice. We examined fasting blood glucose (**A**) and serum insulin (**B**) levels in transgenic mice overexpressing mutant p85β and wild-type p85β. Data are means ± s. e. m. *P<0.05, **P<0.01, t-test.

**Supplementary Fig. 6**


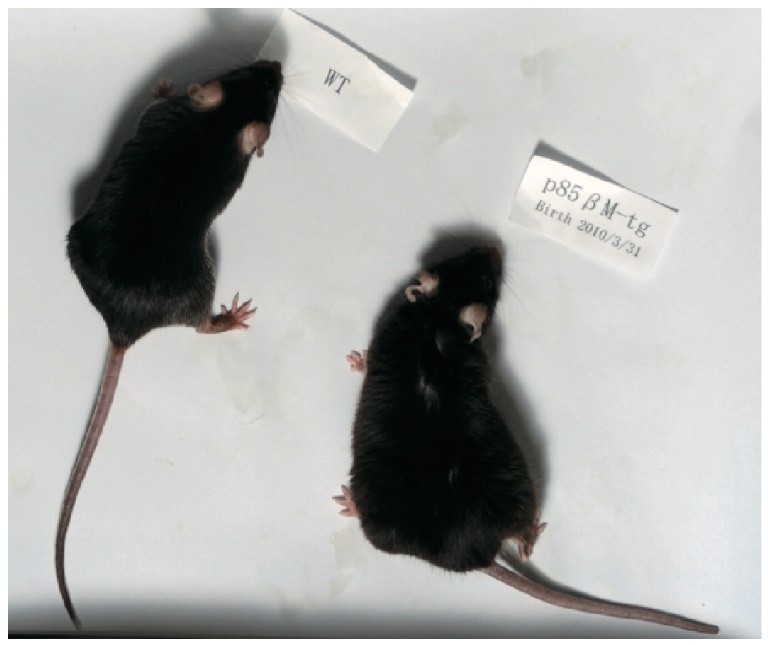


Mutant p85β transgenic male mouse (p85βM-tg) with a prolonged lifespan. The mice showed a prolonged lifespan of 179 days over the average lifespan of control mice.

**Supplementary Fig. 7**

PDGF receptor associated more stably with mutant p85β protein than with wild-type p85β. Growth factor-specific receptor phosphorylation in the PDGF receptor (PDGFR) and insulin receptor (insulin-R) was observed in normal human diploid fibroblasts (NHDFs), normal human diploid fibroblast clone cells overexpressing wild-type p85β (NHDFWT1) and normal human diploid fibroblast clone cells overexpressing mutant p85β (NHDFM1) (**A**). To examine the association of p85β protein with the PDGF receptor (PDGFRβ) and insulin receptor substrate protein (IRS-1), FLAG-epitope-tagged wild-type p85β- and mutant p85β-transfected normal human diploid fibroblast clone cells of NHDFM1 and NHDFWT1 were serum-starved and incubated with PDGF (8 ng/ml) for 10 min (**B** and **D**) or with insulin (100 ng/ml) for 5 min **(C**). Ligand was removed and the cells were incubated for 2 or 4 hours before lysis. Lysates were subjected to immunoprecipitation with an anti-p85β antibody **(B**), anti-IRS-1 antibody **(C**) or anti-PDGFRβ antibody **(D**). Binding of PDGFRβ or p110α protein to p85β protein **(B**), binding of p85α protein to IRS-1 **(C**) and binding of p85α protein or p85β protein to PDGFRβ **(D**) were detected by Western blot analysis with PDGFRβ-, p110α- or p110β-antibody **(B**) and with p85α- or p85β-(anti-FLAG)-antibody (**C** and **D**). However, binding of p110β protein to p85β protein **(B**) and binding of p85β protein to IRS-1 **(C**) were not detected by the same analysis (data not shown).

**Supplementary Fig.8**


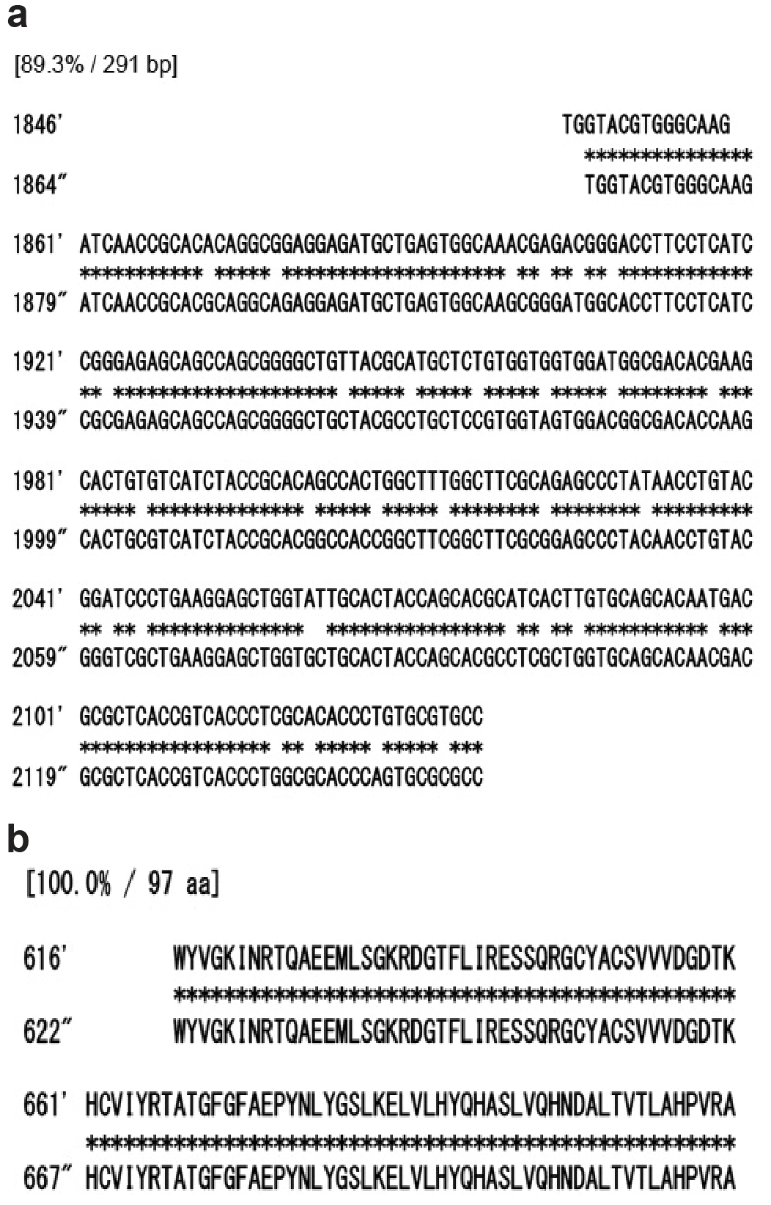


Comparison of C-SH2 domain nucleotide and amino acid sequence homologies between rat p85β and human p85β. (**A**) Both the rat and human nucleotide sequences of p85β measured 321 bases, with 36 differences (**B**) Both the rat and human amino acid sequences of p85β consisted of 107 amino acids with identical sequences.

**Supplementary Fig. 9**


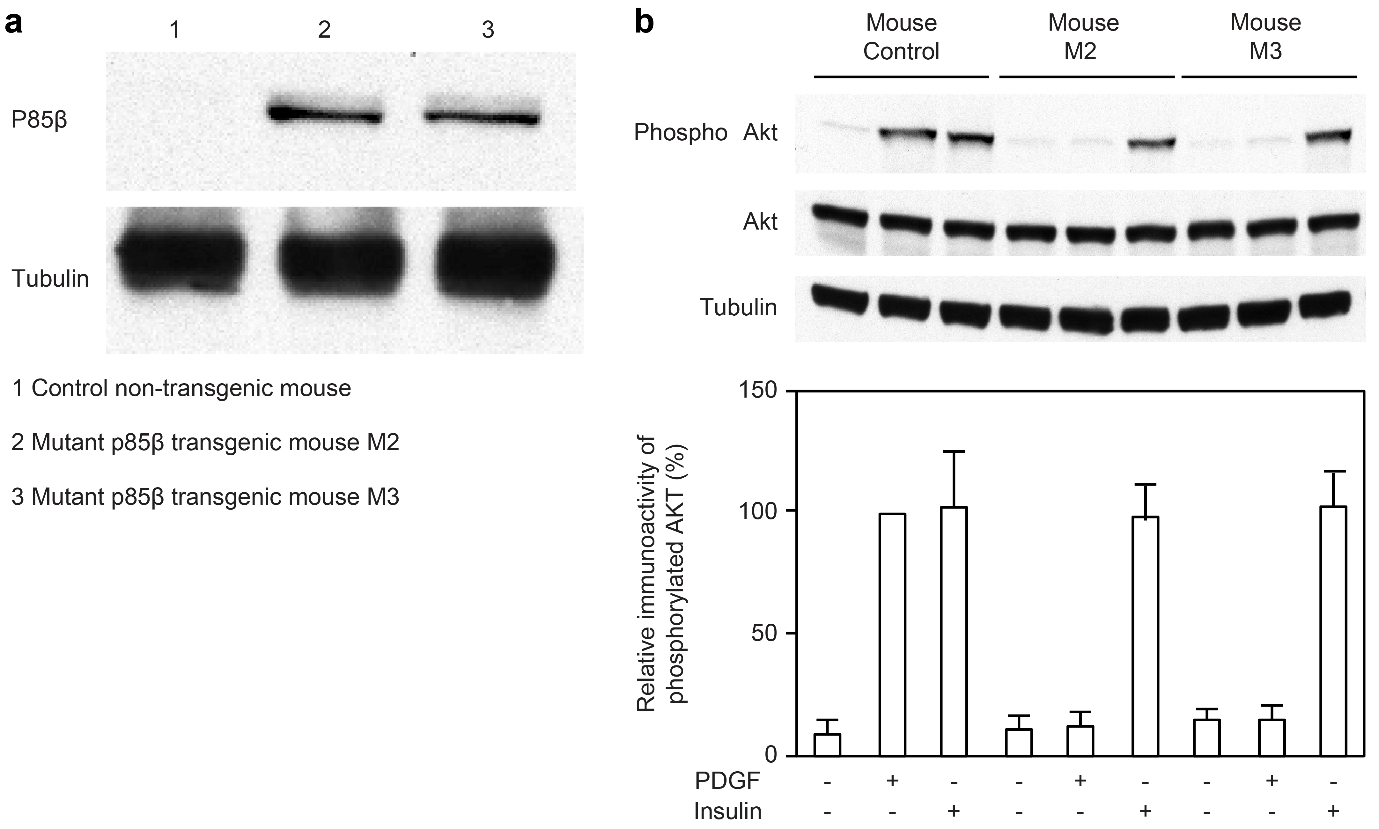


The p85β mutant gene regulates PI3K/Akt pathways of transgenic mouse cells with a prolonged lifespan.

(**A**) Protein expression levels of FLAG-epitope-tagged mutant p85β transgenes of primary skin fibroblasts from transgenic mice. 1 indicates a control non-transgenic mouse. 2 and 3 indicate two mutant-type p85β transgenic mice (mice M2 and M3, respectively). The expression of p85β protein was detected using anti-FLAG DDDK antibody. (**B**) PDGF-induced-Akt phosphorylation in primary skin fibroblasts from two mutant-type p85β transgenic mice (M2 and M3) with a prolonged lifespan was examined. Data are means ± s. e. m.

**Supplementary Fig. 10**


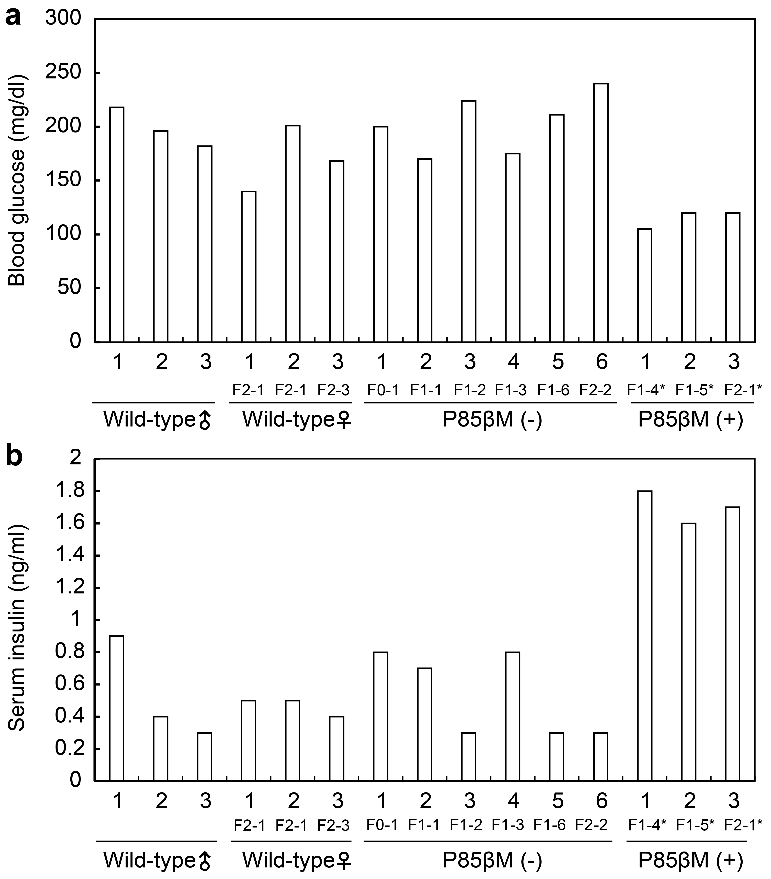


Fasting blood glucose and insulin levels in transgenic mice. We examined fasting blood glucose (**A**) and serum insulin (**B**) levels in mutant p85β transgenic mice. ♂ mice 1-3 are of wild-type No 1, 2 and 3. ♀ mice 1-3 are of wild-type F2 No 1, 2 and 3. p85β M (-) mice 1-6 are mice of p85β M F0 No 1, p85β M F1 No 1, 2, 3, 6 and F2 No 2. p85β M (+) mice 1-3 are of p85β M F1 No 4*, 5* and F2 No 1* shown in Table 2.

**Supplementary Fig. 11**


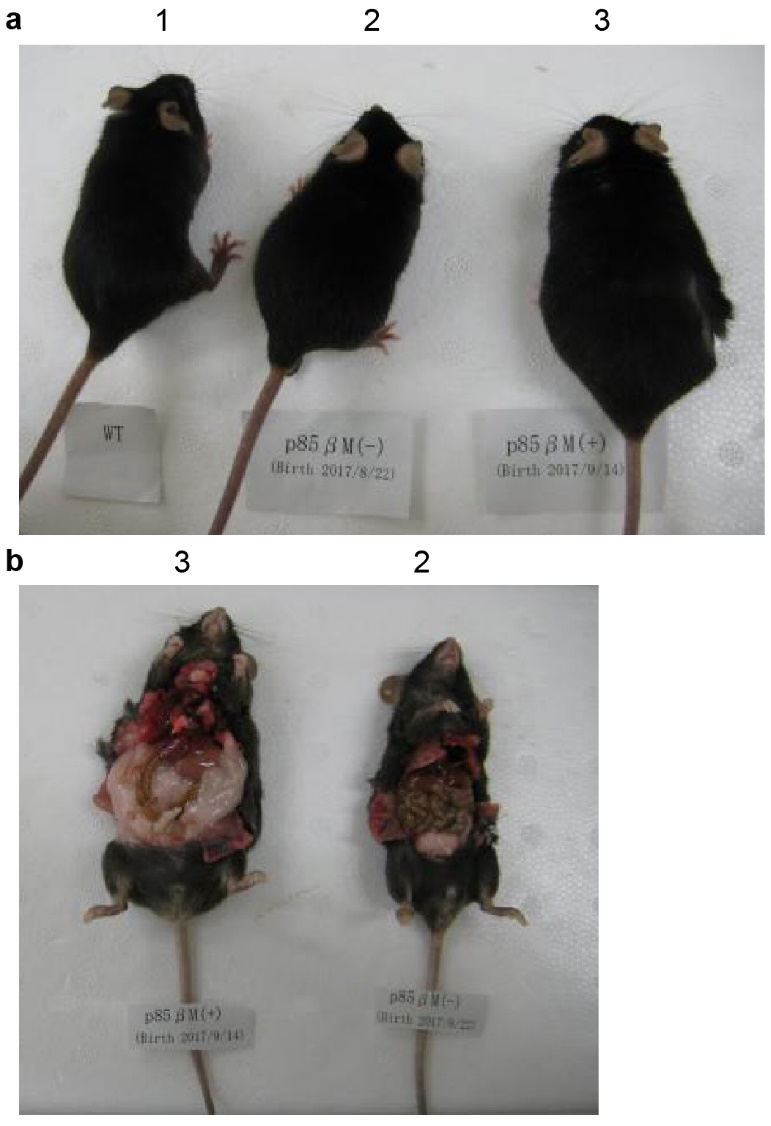


Mutant p85β transgenic mice (p85βM) with visceral fat.

A mouse No3 is a candidate mouse p85βM (+) that may have a prolonged lifespan from a mutant p85β gene (A). This mouse shows extremely high visceral fat (B). Despite the transfection of the p85β gene in a No 2 mouse, it is considered that there is no prolonged lifespan due to a normal level of insulin. No 2 and No 3 mice are of p85βM No 3 and p85βM No 5* shown in Table 2, respectively.

**Supplementary Fig. 12**


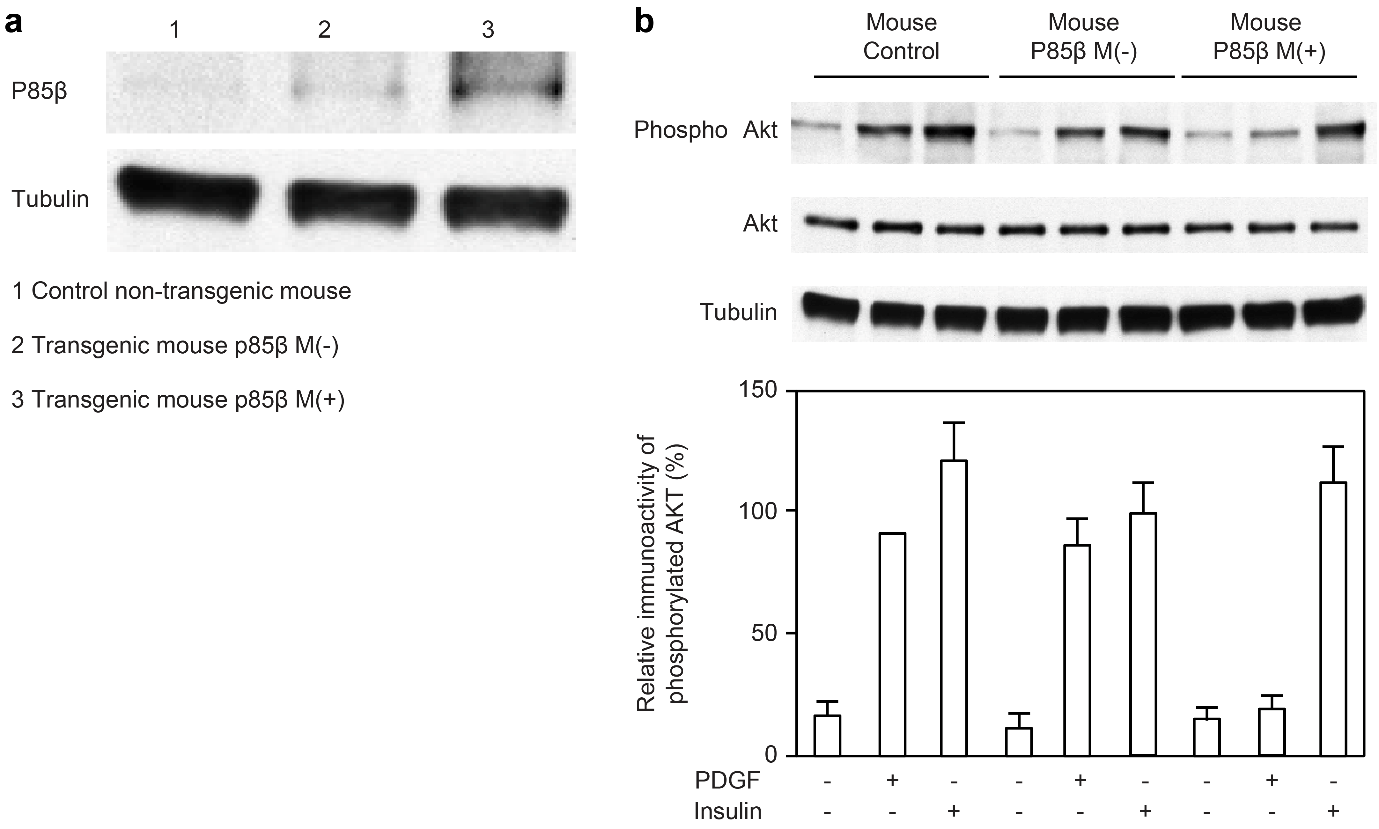


The p85β mutant gene regulates PI3K/Akt pathways of transgenic mouse cells.

(**A**) Protein expression levels of FLAG-epitope-tagged mutant p85β transgenes of primary skin fibroblasts from transgenic mice. 1 indicates a control non-transgenic mouse. 2 indicates a mutant-type p85β transgenic mouse (p85βM (-)). This mouse is considered not to have a prolonged lifespan due to low levels of mutant p85β protein. 3 indicates a mutant-type p85β transgenic mouse (p85βM (+)). This mouse is considered a candidate for a prolonged lifespan due to low levels of serum insulin. 2 and 3 mice are of p85βM F1 No 2 and p85βM F2 No 1, respectively, shown in Table 2. (**B**) PDGF-induced-Akt phosphorylation in primary skin fibroblasts from two mutant-type p85β transgenic mice p85βM (-) and p85βM (+), respectively. Data are means ± s. e. m.
